# c-Fos over-expression promotes radioresistance and predicts poor prognosis in malignant glioma

**DOI:** 10.18632/oncotarget.11779

**Published:** 2016-09-01

**Authors:** Zhi-Gang Liu, Guanmin Jiang, Jiao Tang, Hui Wang, Guokai Feng, Furong Chen, Ziwei Tu, Guiyun Liu, Yu Zhao, Ming-Jing Peng, Zheng-Wen He, Xiao-Yan Chen, Holly Lindsay, Yun-Fei Xia, Xiao-Nan Li

**Affiliations:** ^1^ Key Laboratory of Translational Radiation Oncology, Hunan Province, Department of Radiotherapy, Hunan Cancer Hospital, The Affiliated Cancer Hospital of Xiangya School of Medicine, Central South University, Changsha, 421001, P.R. China; ^2^ Department of Clinical Laboratory, Hunan Cancer Hospital, The Affiliated Cancer Hospital of Xiangya School of Medicine, Central South University, Changsha, 421001, P.R. China; ^3^ State Key Laboratory of Oncology in Southern China, Sun Yat-sen University Cancer Center, Guangzhou, 510060, P.R. China; ^4^ Translational Medicine Center, Hunan Cancer Hospital, The Affiliated Cancer Hospital of Xiangya School of Medicine, Central South University, Changsha, 421001, P.R. China; ^5^ Department of Neurosurgery, Hunan Cancer Hospital, The Affiliated Cancer Hospital of Xiangya School of Medicine, Central South University, Changsha, 421001, P.R. China; ^6^ Department of Pathology, Hunan Cancer Hospital, The Affiliated Cancer Hospital of Xiangya School of Medicine, Central South University, Changsha, 421001, P.R. China; ^7^ Laboratory of Molecular Neuro-Oncology, Texas Children's Cancer Center, Baylor College of Medicine, Houston TX, 77030, USA

**Keywords:** malignant glioma, radioresistance, c-Fos, prognosis

## Abstract

*c-Fos* is a major component of activator protein (AP)-1 complex. It has been implicated in cell differentiation, proliferation, angiogenesis, invasion, and metastasis. To investigate the role of *c-Fos* in glioma radiosensitivity and to understand the underlying molecular mechanisms, we downregulated *c-Fos* gene expression by lentivirus-mediated shRNA in glioma cell lines and subsequently analyzed the radiosensitivity, DNA damage repair capacity, and cell cycle distribution. Finally, we explored its prognostic value in 41 malignant glioma patients by immunohistochemistry. Our results showed that silencing *c-Fos* sensitized glioma cells to radiation by increasing radiation-induced DNA double strand breaks (DSBs), disturbing the DNA damage repair process, promoting G2/M cell cycle arrest, and enhancing apoptosis. *c-Fos* protein overexpression correlated with poor prognosis in malignant glioma patients treated with standard therapy. Our findings provide new insights into the mechanism of radioresistance in malignant glioma and identify *c-Fos* as a potentially novel therapeutic target for malignant glioma patients.

## INTRODUCTION

Glioblastoma (GBM) is the most common adult primary brain tumor, accounting for approximately 40% of all central nervous system malignancies. The current standard of treatment for newly-diagnosed glioblastoma is surgical resection followed by radiotherapy plus concomitant adjuvant temozolomide [1]. The prognosis of GBM is poor, with a median survival of only 12-15 months [2]. One and five year survival rates are less than 20% and 5%, respectively [3]. Resistance to ionizing radiation is one of the most important causes of the poor prognosis of this deadly disease [4]. Thus, it is imperative to conquer the resistance of human glioma cells to radiotherapy to improve the survival of malignant glioma.

The JNK stress pathway is a member of the mitogen-activated protein kinase (MAPK) superfamily and includes c-Jun N-terminal protein kinase (JNK)/stress-activated protein kinase (SAPK), p38, and extracellular signal-regulated kinase (ERK). It participates in multiple intracellular processes including cell growth, differentiation, transformation, and apoptosis by increasing expression of activating protein-1 (AP-1) [5]. As a major component of AP-1 complex, *c-Fos* has been implicated in signal transduction, cell differentiation proliferation, cell motility, cancer growth, angiogenesis, invasion, and metastasis [6–8]. Recent studies have identified *c-Fos* as one of the early response genes toward ionizing radiation [9]. Together with c-jun, Egr-1, and NFκB, the induced expression of *c-Fos* by radiation triggered a series of downstream genes important in the adaptation of cells and tissues to radiation-induced stress [7, 10]. Additionally, *c-Fos* induction was observed even in cells treated with low radiation doses (0.5 to 2 Gy) [11], although this induction was transient, reaching a maximum level at 1 h and declining to the constitutive level by 4 hours [12]. Conversely, mouse fibroblast cell line deficient in *c-Fos* (*c-Fos* −/−) was found to be more sensitive to radiation, demonstrating increased cell death and apoptosis [13, 14]. An increase in AP-1 DNA-binding activity was associated with increased cellular resistance to cancer therapeutic agents [15]. Altogether, previous studies suggested that *c-Fos* may play an important role in cellular responses toward ionizing radiation.

Many studies have been reported correlating *c-Fos* expression with clinical prognosis. The conclusions, however, were mixed. In human squamous cell lung carcinoma [16], breast carcinoma [17], human osteosarcoma [18], oral squamous cell carcinoma [19], and cutaneous squamous cell carcinoma [20], *c-Fos* overexpression was found to correlate with poor prognosis; while in refractory colorectal carcinoma [21] and epithelial ovarian carcinoma [22], elevated *c-Fos* expression was reported to be a good prognostic marker. There were additional studies from large numbers of patients with gastric cancer showed that loss of *c-Fos* expression correlated with shorter survival, advanced stage, lymph node metastasis, and lymphatic invasion [23, 24]. Understanding the prognostic value of *c-Fos* expression in human malignant gliomas, which remains unclear, is therefore needed.

In this study, we tested our hypotheses that *c-Fos* plays a critical role in converting extracellular signals into gene expression changes in order to prepare GBM cells to radiation-induced stress and subsequent development of radioresistance and targeting *c-Fos* may improve radiosensitivity. We investigated the contribution of *c-Fos* to radiosensitivity in glioma cells and analyzed its underlying mechanisms, including DNA damage repair capacity, cell cycle distribution, and related protein expression. We also determined if *c-Fos* expression is correlated with the clinical outcomes in malignant gliomas.

## RESULTS

### *c-Fos* silencing inhibited human glioblastoma cell viability

**Figure 1:**
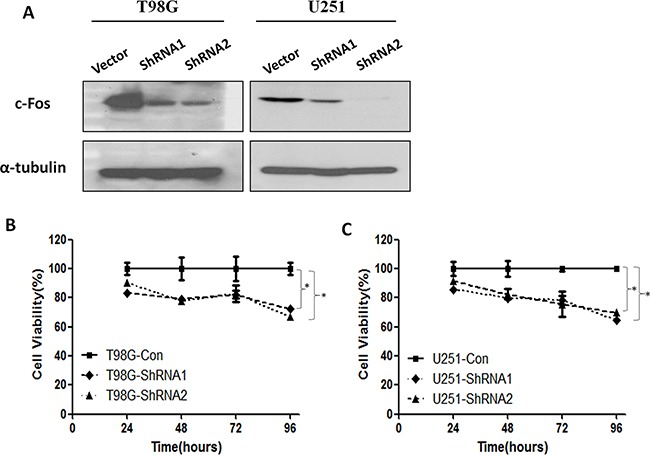
*c-Fos* expression and cell viability were inhibited by ShRNA **A.** Western blot analysis performed on control vector and *c-Fos* shRNA using anti-*c-Fos*, and anti-α-tubulin antibodies. **B.** T98G glioma cell viability was inhibited by ShRNA1 and ShRNA2. *P<0.05; t-test. **C.** The viability of U251 glioma cells was inhibited by ShRNA1 and ShRNA2. *P<0.05; t-test.

To functionally demonstrate the importance of *c-Fos* in radiation responses of malignant gliomas, we used a targeting approach based on lentivirally expressed shRNAs (LV-shRNA) to knockdown *c-Fos* mRNA. First, we verified whether T98G and U251 cells, well-established GBM cell lines, were successfully transfected with LV-shRNA by immunoblotting. As shown in Figure 1A, in both T98G and U251 cells, the relative density of *c-Fos* in both shRNA1-infected cells and shRNA2-infected cells was significantly decreased compared with control group cells, confirming that LV-shRNA effectively silenced *c-Fos* in both cell lines. Since the silencing was more effective in shRNA2-treated cells, we chose the shRNA2 as an optimal silencing method in the subsequent assays.

We next examined whether *c-Fos* silencing could inhibit cell viability in GBM cell lines (Figure 1B and 1C). In T98G cells, *c-Fos* silencing markedly reduced cell viability, reaching a maximum inhibition (Cell viability in Day 4=66.9%) as indicated by the decrease in optical density levels (Figure 1B, P<0.05). In U251 glioblastoma cells, *c-Fos* silencing also reduced cell viability, reaching a maximum inhibition (Cell viability in Day 4=64.5%) in Day 4 (Figure 1C, P<0.05). In addition, curves from both T98G and U251 cells revealed that there were no significant differences in cell viability between the shRNA1 or shRNA2 treatment groups.

### *c-Fos* silencing increased human glioblastoma cells radiosensitivity

**Figure 2:**
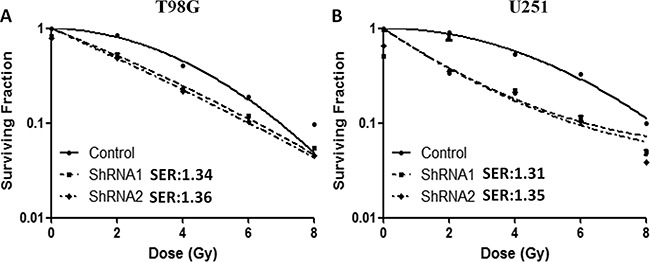
*c-Fos* regulated radiosensitivity in T98G and U251 cell lines **A.** Down-regulation of *c-Fos* increased the sensitivity of T98G cells to radiation. P<0.05; ANOVA test. **B.** Down-regulation of *c-Fos* increased the sensitivity of U251 cells to radiation. P<0.05; ANOVA test.

Next, we investigated whether *c-Fos* silencing was able to increase the sensitivity of glioblastoma cells to radiation. For T98G and U251 cells, both shRNA1-treatment (SER=1.34 and 1.31, respectively) and shRNA2-treatment (SER=1.36 and 1.35, respectively) enhanced radiosensitivity (Figure 2, P<0.05, ANOVA test; SF2=0.49 for T98G *c-Fos* ShRNA cells, SF2=0.33 for U251 *c-Fos* ShRNA cells). As SER=1 suggests an additive radiation effect, SER >1 a supra-additive effect, and SER<1 a sub-additive effect. Thus, SER=1.43, more than 1, means *c-Fos* silence could increase the radiosensitivity to radiation. These data suggested that *c-Fos* may be a critical regulator of radiation response in glioblastoma cells.

### *c-Fos* silencing enhanced radiation-induced G2/M cell cycle arrest, radiation-induced DNA double strand breaks and suppressed DNA repair

**Figure 3:**
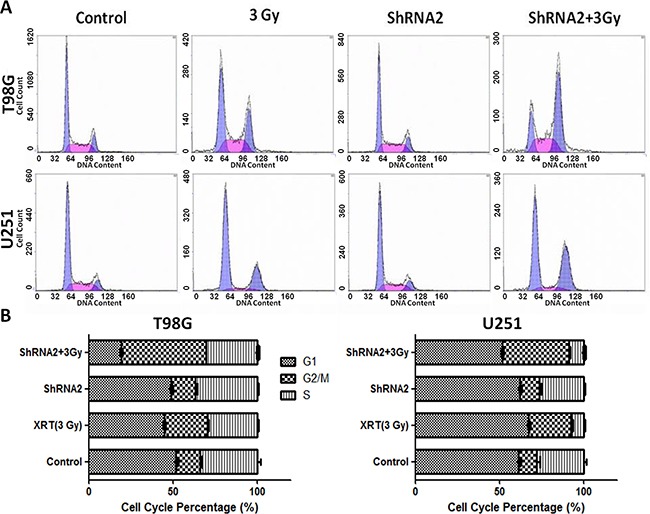
*c-Fos* depletion combined with 3 Gy radiation increased the sub-G2/M population **A.** Analysis of cell cycle distribution by flow cytometry. **B.** Sub-population percentage analysis by flow cytometry for T98G cell line. **C.** Sub-population percentage analysis by flow cytometry for U251 cell line.

Cell cycle regulation is thought to be the foremost determinant of ionizing radiation sensitivity. Thus, we used flow cytometric analysis to determine the effect of *c-Fos* silencing. For both T98G and U251 cells, ionizing radiation at 3 Gy resulted in significantly increase of G2/M fractions in cells with *c-Fos* knockdown (Figure 3). There are significant differences among the treatment groups(P<0.001, One Way ANOVA, Holm-Sidak method), except for control group and ShRNA2 group.

**Figure 4:**
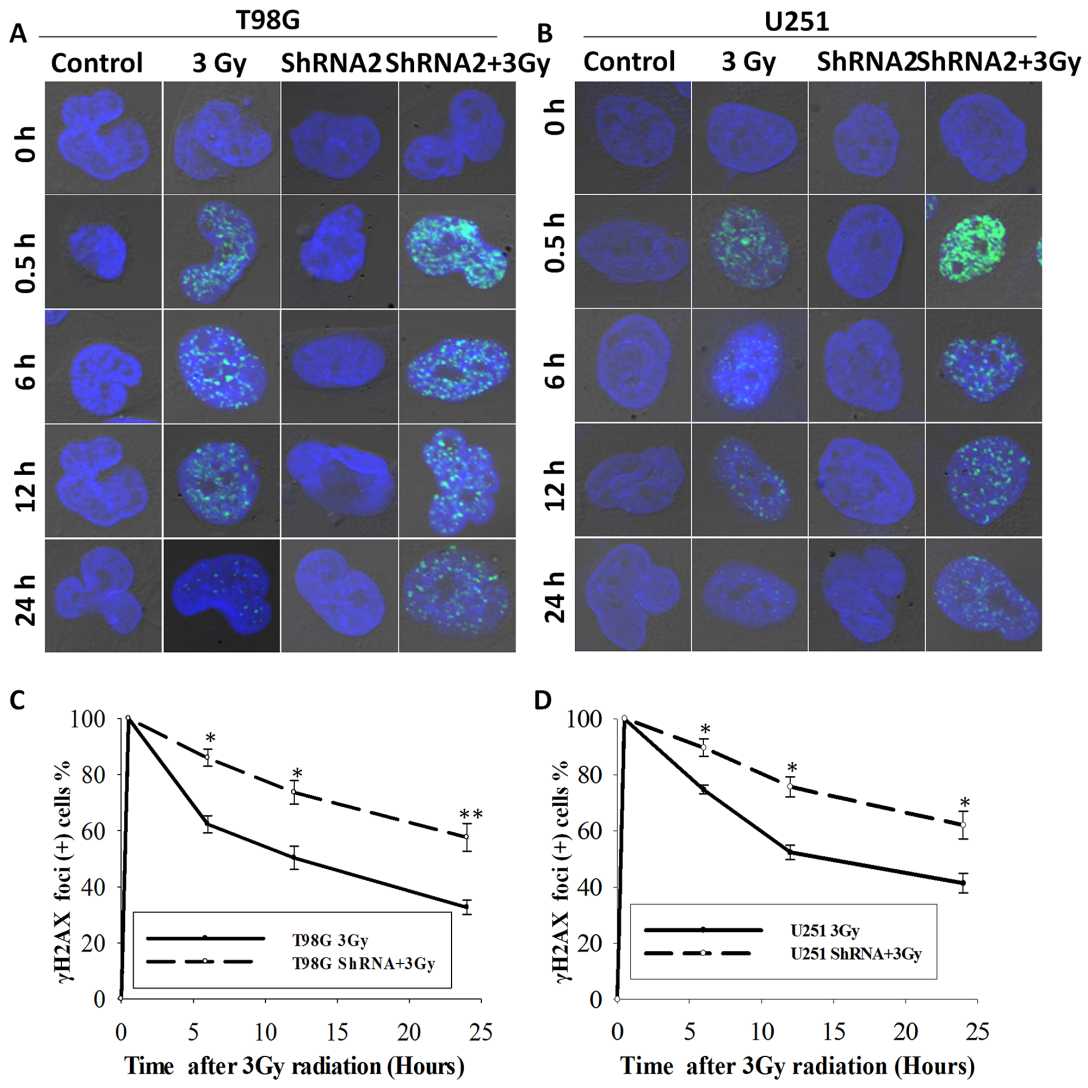
*c-Fos* knockdown delayed DNA damage repair in T98G and U251 cell line **A.** γH2AX foci in *c-Fos*-depleted T98G cells vs. control at various time points following 3 Gy radiation. Original magnification, x600. **B.** γH2AX foci in *c-Fos*-depleted U251 cells vs. control at various time points following 3 Gy radiation. Original magnification, X600. **C.** γH2AX foci positive cells at various time points following 3 Gy radiation in both T98G-3Gy cells and T98G-ShRNA2+3Gy cells. *P<0.05; **P<0.01, t-test. **D.** γH2AX foci positive cells at various time points following 3 Gy radiation in both U251-3Gy cells and U251-ShRNA2+3Gy cells. *P<0.05, **P<0.01, t-test.

Next, we evaluated the DNA damage responses (DDR) using the well-established marker of DNA double strand breaks (DSB) γH2AX, a phosphorylated form of H2AX at Ser139 (γH2AX). In tumor cells treated with and without shRNA2, a time-course analyses of γH2AX kinetic changes were performed before and after radiation (0.5 h, 6 h, 12 h, and 24 h) through immunofluorescent staining. As shown in Figure 4, depletion of *c-Fos* did not cause a significant difference in DSB levels compared to non-irradiated control cells or 0.5 h after radiation. However, a significant increase of γH2AX positive cells were observed in *c-Fos* knockdown cell lines 6 h, 12 h, and 24 h after administration of 3 Gy radiation. In T98G glioma cells, 30 minutes after administration of 3 Gy radiation, almost 100% of cells in both the control and the *c-Fos* knockdown groups retained γH2AX foci. At 6 h, foci remained in only 62.3% of control cells compared to 87.0% of *c-Fos* knockdown cells(P=0.007; t-test). At 12 h, foci persisted in only 50.8% of control cells compared to 72.6% of *c-Fos* knockdown cells (P=0.0023; t-test). After 24 h, the number of γH2AX positive foci in control cells and *c-Fos* knockdown cells was 32.2% and 58.7%, respectively (P=0.0014; t-test). Similarly, in U251 glioma cells, we found that *c-Fos* depleted cells maintained γH2AX foci longer than control cells in 24h(P=0.00042; t-test).

These data showed that the loss of *c-Fos* not only affected the induction of γH2AX foci but also resulted in a disruption of DSB repair leading to significant prolongation of γH2AX foci formation after exposure to ionizing radiation. They indicated that *c-Fos* knockdown sensitized GBM cell lines to radiation by decreasing DSB repair capacity.

### *c-Fos* silencing altered the expression of cell cycle-related protein and induced cellular apoptosis

**Figure 5:**
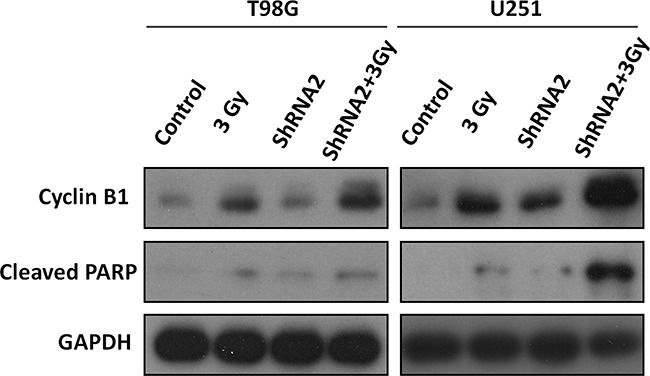
Western blot analysis of cell cycle and apoptosis-related proteins T98G-3Gy cells, T98G-ShRNA+3Gy cells, U251-3Gy cells, and U251-ShRNA+3Gy cells were harvested 48 hours after 3 Gy radiation. Lysates were subjected to Western blot analysis with the labeled antibodies.

We next examined the expression of cell cycle-related proteins. Figure 5 shows that CyclinB1 expression were significantly dysregulated after administration of 3 Gy ionizing radiation to T98G and U251 cells and that depletion of *c-Fos* exacerbated this increase. Figure 5 also showed that *c-Fos* silencing followed by radiation induced the expression of cleaved-PARP, the final product of the apoptotic state [25], in both cell lines, especially in U251 cells.

### *c-Fos* overexpression inversely correlated with the survival time of patients with malignant glioma

**Figure 6:**
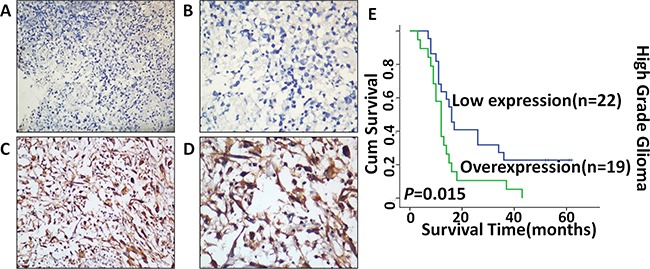
Expression of *c-Fos* in malignant glioma tissues detected by immunohistochemistry staining and Kaplan–Meier estimates of survival probability Negative staining of *c-Fos* in glioma tissue **A.** 200×, **B.** 400×; strong staining of *c-Fos* in nucleus and cytoplasm **C.** 200×, **D.** 400×; High *c-Fos* expression levels were significantly associated with poor overall survival (P=0.015) in malignant glioma patients **E.**

Next, we explored whether *c-Fos* overexpression correlated with the prognosis of malignant glioma patients, and clinical characteristics of WHO Grade III and IV gliomas were presented in Table 1. As shown in Figure 6(A-D), *c-Fos* expression was primarily localized to the nucleus and cytoplasm of tumor cells and was observed in 80.5% (33/41) of the analyzed patients. The survival curve demonstrated that overexpression of *c-Fos* predicted shorter overall survival time for WHO Grade III and IV gliomas (Figure 6E). Mean overall survival times in the *c-Fos* high expression group and low expression group were 13.9 months and 37 months (P= 0.015), respectively. Thus, *c-Fos* expression was inversely correlated with the survival rate of human high-grade glioma patients.

**Table 1:** Clinical characteristics of WHO Grade III and IV gliomas

Characteristics	All patients (n=41)	c-Fos Low expression (n=22)	c-Fos High expression (n=19)	P value (Chi-square test)
Age(years)				
Mean	43.9±16.2	47.4±16	39.9±16	
Median	40	54	37	
Range	12~72	15~72	12~68	
KPS				
≥70	39	21	18	1.000
<70	2	1	1	
Gender				
Male	28	16	12	0.511
Female	13	6	7	
WHO				
III	27	14	13	0.747
IV	14	8	6	

## DISCUSSION

In this report, we presented the first evidence that *c-Fos* expression was correlated with radiation response in human glioma cell lines. *c-Fos* silencing sensitized glioma cells to radiation by inhibiting cell viability, disturbing DNA damage repair, promoting G2/M cell cycle arrest, and enhancing cellular apoptosis. In addition, we also discovered an inverse correlation between *c-Fos* expression and malignant glioma patients' survival.

In terms of the radiosensitizing effects of *c-Fos*, our data were consistent with an earlier study which showed that *c-Fos* knockdown cells are hypersensitive to the cytotoxic effect of UV light, including UV-induced chromosomal mutations and DNA breakage [26]. We further explored the mechanisms of the increased radiosensitivity caused by *c-Fos* depletion. We showed that *c-Fos* silencing alone could inhibit human glioma cell viability, which is consistent with results from human colon carcinoma [27] and bladder cancer cells [28, 29], in which it was also shown that *c-Fos* knockdown suppresses cell growth.

DNA double strand breaks are the primary mechanism of tumor cell death following radiation, and radiation causes DNA damage either directly or indirectly through regulation of cell cycle checkpoints [30]. Such regulation on cell cycle progression may be another important mechanism for radiosensitization, as supported by our results that *c-Fos* disruption increased cell-cycle arrest (Figure 3). The Cyclin B is the main target molecule of the G2/M cell cycle checkpoint [31, 32]. Our results showed that *c-Fos* knockdown upregulated CyclinB1 expression in GBM cell lines after radiation compared to control cells. However, further studies are needed to determine the exact mechanism for the G2/M arrest induced by *c-Fos* knockdown. As G2/M phase is the most radiosensitive phase of the cell cycle, it may account in part for the effects of *c-Fos* deficiency on the enhancement of radiation sensitivity in T98G and U251 GBM cell lines. Another study showed that abundance of cyclin G1 increased radiosensitivity of cancer cells through activation of cyclin B1, enhanced radiosensitivity was correlated with increased cyclin B1 [33]. It could be another mechanism of *c-Fos* inhibition radiosensitized GBM cells. Our results also indicated that *c-Fos* knockdown increased the persistence of γH2AX foci positive cells 24 h after radiation (Figure 4), which indicated the delayed DNA damage repair of DSBs. The role of *c-Fos* on activation or inhibition of apoptosis in cancer cells remains controversial. *c-Fos* was shown to enhance both p53-dependent and p53-independent steroid-induced apoptosis in T-lymphocytes [34]. However, many other studies conflicted with the above findings. For example, *c-Fos* protein down-regulation promotes apoptosis in cervical cancer cells through inhibition of ERK1/2 [35]. In addition, another study demonstrated that *c-Fos* decreased P-glycoprotein expression and activity and altered expression of apoptosis-associated proteins (i.e., Bax, Bcl-2, p53, and PUMA) in the MCF-7 breast cancer cell line [36]. In the present study, our data in T98G and U251 GBM cell lines also supported the finding that *c-Fos* silencing enhances cancer cell apoptosis.

In terms of the correlation between *c-Fos* expression and cancer prognosis, *c-Fos* has been shown to play different, even opposite, roles in different cancers. In gastric cancers, elevated *c-Fos* expression is a good prognostic marker for patients, These findings may be attributed to the fact that *c-Fos* expression has tumor suppressive activity in gastric cancer, possibly related to its pro-apoptotic function [23, 24]. Contrary to the above results, however, a recent study in 333 patients with pancreatic cancer showed that high *c-Fos* expression was a significant marker of poor overall survival [37]. Overexpression of *c-Fos* promotes cell invasion and migration via the CD44 pathway in oral squamous cell carcinoma [19], and may contribute to the metastasis and cell migration of human hepatocellular carcinoma [38]. Additionally, knockdown of *c-Fos* suppresses the migratory behavior of human colon carcinoma cells by blockade of TGFβ1 production in athymic mice [39]. Our data from high-grade glioma patients support the inverse relationship between *c-Fos* overexpression and tumor prognosis. We hypothesize that the conflicting correlation between *c-Fos* expression and prognosis in various cancer types may be attributed to the varying effects of *c-Fos* on tumor cell apoptosis as described above; this may be secondary in part to organ-specific functions of *c-Fos*.

In conclusion, we showed that *c-Fos* silencing sensitizes GBM cells to radiation by increasing radiation-induced DNA DSB, disturbing DNA damage repair, and regulating G2/M cell cycle and apoptosis. Additionally, *c-Fos* overexpression is correlated with poor survival for malignant glioma patients. Our results suggested that *c-Fos* may serve as a novel molecular maker of malignant glioma and a possible novel therapeutic target for radiosensitizing in GBMs. Our results justified the initiation of preclinical testing of targeted therapies against *c-Fos* in malignant gliomas.

## MATERIALS AND METHODS

### Cell lines and treatments

T98G and U251 human glioblastoma cell lines were obtained from the American Type Culture Collection (ATCC, Manassas, VA) and incubated in DMEM (Invitrogen, Carlsbad, CA) supplemented with 10% fetal bovine serum (FBS; Hyclone, Logan, UT), penicillin (100 units/ml), and streptomycin (100 units/ml) at 37°C in a humidified 5% CO2 incubator. Double stranded oligonucleotides with homology to a desired target region of human *c-Fos* were synthesized by Guangzhou RiboBio Co., Ltd. (Guangzhou, China) and the target sequence was GCAAGGTGGAACAGTTATC. Negative control siRNA was supplied by Guangzhou RiboBio Co., Ltd. T98G. U251 cells were transfected with plasmids containing shRNA1 (5′GCAAGGUGGAACAGUUAUCdTdT3, 3′dT dTCGUUCCACCUUGUCAAUAG5, siB0849110500) or shRNA2(5′UGCCAGACAUGGACCUAUCdTdT3, 3′dTdT ACGGUCUGUACCUGGAUAG5, siB0849110519) using Lipofectamine 2000 (Invitrogen). Positive transfectants were selected by incubating cells with 0.8 mg/ml G418 (GIBCO BRL) for two weeks to obtain a stable cell line for *c-Fos* silencing in subsequent assays. Transduction efficiency was determined by western blotting.

### Cell viability

We analyzed T98G and U251 cell viability after *c-Fos* silencing. Cell viability was measured with the Cell Counting Kit (CCK-8) assay. Briefly, after exposing T98G and U251s cell to *c-Fos* silencing for 1-4 days, 20 μL of CCK-8 (Sigma, St. Louis, MO) dissolved in phosphate-buffered saline (PBS) at a concentration of 5 mg/mL was added to the cancer cells and incubated in a CO2 incubator for 3 hours. The medium was then aspirated and the absorbance of each well measured using plate reader at a test wavelength of 460 nm with a reference wavelength of 630 nm. Optical density (OD) was utilized as the indicator of cell survival.

### Survival fraction assay

Clonogenic survival assays was performed as described previously [40]. Briefly, 500 cells were seeded onto 60-mm dishes. After 4 hours, T98G–shRNA1, T98G–shRNA2, U251-shRNA1, U251-shRNA2 were radiated with different doses of ionizing radiation (0, 2, 4, 6 and 8 Gy) with a 210kV X-ray source at 2.16 Gy/min (RS-2000 Biological irradiator, Rad Source Technologies, Alpharetta, GA). After being cultured in a 37°C, 5% CO2 incubator for 10-14 days, the plates were stained with 0.5% crystal violet. The number of colonies were then determined and the surviving fractions, plating efficiency (PE), and survival fractions (SF) were calculated. Survival curves were fitted and analyzed using a linear-quadratic model [S=exp (−αD-βD2)] by GraphPad Prism software (version 4.0, GraphPad Prism software, San Diego, CA). The radiation sensitizing enhancement ratio (SER) by *c-Fos* silence was calculated using the following formula: SER= (SF2 of control)/(SF2 of *c-Fos* silencing), SF2: surviving fraction at 2 Gy; SER=1 suggests an additive radiation effect, SER >1 a supra-additive effect, and SER<1 a sub-additive effect.

### Determination of cell cycle

FACS (fluorescence activating cell sorter) analysis was used to determine cell cycle distribution of the GBM cell lines following radiation. The cell suspension was prepared by trypsinization, and 1 × 106 cells/mL were washed twice with PBS. The cells were re-suspended with 10 mL of 70% ethanol (−20°C), incubated at 4°C for 4 h, washed twice in cold PBS, and incubated with RNase (Sigma) at a concentration of 0.25 mg/mL at 37°C for 15 min. The suspension was then treated with PI (10 μL/mL) and incubated for 15 min at 4°C in the dark. DNA histograms were analyzed using same FACS machine to evaluate the cell cycle distribution.

### Immunofluorescent analysis of γH2AX foci

Cells grown in chamber slides were fixed and permeabilized. They next were incubated with antibody to phospho-H2AX (Millipore) followed by goat-anti-mouse-Alexa488 (Invitrogen) then and mounted with Prolong gold anti-fade reagent containing 40, 6-diamidino-2-phenylindole (DAPI; Invitrogen) to visualize nuclei. Slides were then examined by fluorescence microscopy (Carl Zeiss Axioskop 2, Thornwood, NY). Cells were judged as ‘positive’ for γH2AX foci when they displayed 10 or more discrete dots of brightness.

### Immunoblotting and antibodies

Cells were grown in 60 mm dishes and treated with 3 Gy radiation, shRNA2, or a combination of both radiation and shRNA2. Cells were washed with ice-cold PBS and scraped into ice-cold lysis buffer (comprised of TRIS-HCl pH 7.8 20 mM, NaCl 137 mM, EDTA pH 8.0 2 mM, NP40 1%, glycerol 10%, NaF 10 mM, Leupeptin 10μg/mL, Na2VO4 200 μmol/L, PMSF 5mM, and Aprotinin (Sigma-Aldrich, MO, USA). Lysates were cleared by centrifugation at 13,000 rpm for 10 min at 4°C, and supernatants removed and assayed for protein concentration using the Pierce BCA bovine serum albumin. Protein was quantified using BCA protein assay (Thermo Scientific), separated by SDS-PAGE, transferred to polyvinylidene difluoride (PVDF;Bio-Rad), and probed with the indicated antibodies. Bands were visualized using Pierce ECL Western Blotting Substrate (Thermo Scientific). Anti- *c-Fos* was purchased from Abcam; Anti-cyclinB1, Anti-cleaved PARP and anti-GAPDH were purchased from Cell Signaling Technology. Donkey-anti-rabbit and sheep-anti-mouse horseradish peroxidase-conjugated secondary antibodies were purchased from GE Healthcare. Images were captured with a FUJIFILM LASS-3000 camera system.

### Human glioma tissue immunohistochemistry and assessment

The human glioma tissue specimens were obtained from 41 malignant glioma patient samples from the Hunan Cancer Hospital in Changsha, China. The study was approved by the Clinical Research Ethics Committee of the Hunan Cancer Center, and written informed consent was obtained from all patients. The pathological grade of these tumors was defined according to the 2007 WHO criteria. Immunohistochemical staining was performed as previously described [39]. In brief, tissue sections with 5 μm thickness were deparaffinized, and endogenous peroxidase was quenched using 3% H2O2 in methanol for 30 min. They were next incubated in a solution of 10% BSA in PBS at 37°C for 1 h to block non-specific binding and were subsequently incubated with IgG (control) or specific antibodies in PBS containing 10% BSA at 4°C overnight. Thereafter, the sections were incubated with a horseradish peroxidase anti-rabbit antibody. Immunohistochemical staining was reviewed by two independent pathologists as previous publications [41]. For evaluation of the staining, the tissues were scored by assessing the *c-Fos* staining in the nucleus. The intensity of staining was graded by an numeric scale that ranged from 0 to 3, representing negative (“0”), weak (“1”), moderate (“2”), and strong (“3”) staining. The number of positive cells were additionally defined as a percentage of the total cell number throughout the entire tissue according to the following scale: 0 for < 5%, 1 for 5–25%, 2 for 26–50%, 3 for 50–75%, and 4 for 75%–100%. The immunostaining final value is the product of intensity and the corresponding value of positive tumor cell percentage for each tumor specimen. Final scores of 0–2 and ≥ 3 were defined as low expression and over-expression, respectively.

### Statistical analysis

For all experiments, the time point was chosen based on pre-experiment results where the most significant effect was detected. Data were expressed as Mean ± SD. Statistical analysis was performed with SPSS version 18. The differences among many groups were compared by One Way ANOVA. Survival curves were analyzed by the method of Kaplan–Meier. Values of P<0.05 were considered statistically significant. All experiments were repeated at least three times.

## References

[1] Stupp R, Mason WP, van den Bent MJ, Weller M, Fisher B, Taphoorn MJ, Belanger K, Brandes AA, Marosi C, Bogdahn U, Curschmann J, Janzer RC, Ludwin SK (2005). Radiotherapy plus concomitant and adjuvant temozolomide for glioblastoma. The New England journal of medicine.

[2] Gurley SN, Abidi AH, Allison P, Guan P, Duntsch C, Robertson JH, Kosanke SD, Keir ST, Bigner DD, Elberger AJ, Moore BM (2012). Mechanism of anti-glioma activity and in vivo efficacy of the cannabinoid ligand KM-233. Journal of neuro-oncology.

[3] Ohgaki H, Dessen P, Jourde B, Horstmann S, Nishikawa T, Di Patre PL, Burkhard C, Schuler D, Probst-Hensch NM, Maiorka PC, Baeza N, Pisani P, Yonekawa Y, Yasargil MG, Lutolf UM, Kleihues P (2004). Genetic pathways to glioblastoma: a population-based study. Cancer research.

[4] Atkins RJ, Ng W, Stylli SS, Hovens CM, Kaye AH (2015). Repair mechanisms help glioblastoma resist treatment. Journal of clinical neuroscience.

[5] Davis RJ (2000). Signal transduction by the JNK group of MAP kinases. Cell.

[6] Milde-Langosch K (2005). The Fos family of transcription factors and their role in tumourigenesis. European journal of cancer (Oxford, England : 1990).

[7] Weichselbaum RR, Hallahan D, Fuks Z, Kufe D (1994). Radiation induction of immediate early genes: effectors of the radiation-stress response. International journal of radiation oncology, biology, physics.

[8] Akagi J, Egami H, Kurizaki T, Ohmachi H, Ogawa M (1997). Signal transduction pathway of the induction of cell motility in hamster pancreatic ductal adenocarcinoma cell. Invasion & metastasis.

[9] Hong JH, Chiang CS, Sun JR, Withers HR, McBride WH (1997). Induction of c-fos and junB mRNA following in vivo brain irradiation. Brain research Molecular brain research.

[10] Gubits RM, Geard CR, Schiff PB (1993). Expression of immediate early genes after treatment of human astrocytoma cells with radiation and taxol. International journal of radiation oncology, biology, physics.

[11] Martin M, Pinton P, Crechet F, Lefaix JL, Daburon F (1993). Preferential induction of c-fos versus c-jun protooncogene during the immediate early response of pig skin to gamma-rays. Cancer research.

[12] Prasad AV, Mohan N, Chandrasekar B, Meltz ML (1995). Induction of transcription of “immediate early genes” by low-dose ionizing radiation. Radiation research.

[13] Lackinger D, Eichhorn U, Kaina B (2001). Effect of ultraviolet light, methyl methanesulfonate and ionizing radiation on the genotoxic response and apoptosis of mouse fibroblasts lacking c-Fos, p53 or both. Mutagenesis.

[14] Lackinger D, Kaina B (2000). Primary mouse fibroblasts deficient for c-Fos, p53 or for both proteins are hypersensitive to UV light and alkylating agent-induced chromosomal breakage and apoptosis. Mutation research.

[15] Bradbury CM, Locke JE, Wei SJ, Rene LM, Karimpour S, Hunt C, Spitz DR, Gius D (2001). Increased activator protein 1 activity as well as resistance to heat-induced radiosensitization, hydrogen peroxide, and cisplatin are inhibited by indomethacin in oxidative stress-resistant cells. Cancer research.

[16] Volm M, Drings P, Wodrich W (1993). Prognostic significance of the expression of c-fos, c-jun and c-erbB-1 oncogene products in human squamous cell lung carcinomas. Journal of cancer research and clinical oncology.

[17] Bland KI, Konstadoulakis MM, Vezeridis MP, Wanebo HJ (1995). Oncogene protein co-expression. Value of Ha-ras, c-myc, c-fos, and p53 as prognostic discriminants for breast carcinoma. Annals of surgery.

[18] Gamberi G, Benassi MS, Bohling T, Ragazzini P, Molendini L, Sollazzo MR, Pompetti F, Merli M, Magagnoli G, Balladelli A, Picci P (1998). C-myc and c-fos in human osteosarcoma: prognostic value of mRNA and protein expression. Oncology.

[19] Dong C, Ye DX, Zhang WB, Pan HY, Zhang ZY, Zhang L (2015). Overexpression of c-fos promotes cell invasion and migration via CD44 pathway in oral squamous cell carcinoma. Journal of oral pathology & medicine.

[20] Zheng Y, Wang GR, Jia JJ, Luo SJ, Wang H, Xiao SX (2014). Expressions of oncogenes c-fos and c-myc in skin lesion of cutaneous squamous cell carcinoma. Asian Pacific journal of tropical medicine.

[21] Singh A, Tong A, Ognoskie N, Meyer W, Nemunaitis J (1998). Improved survival in patients with advanced colorectal carcinoma failing 5-fluorouracil who received irinotecan hydrochloride and have high intratumor C-fos expression. American journal of clinical oncology.

[22] Mahner S, Baasch C, Schwarz J, Hein S, Wolber L, Janicke F, Milde-Langosch K (2008). C-Fos expression is a molecular predictor of progression and survival in epithelial ovarian carcinoma. British journal of cancer.

[23] Jin SP, Kim JH, Kim MA, Yang HK, Lee HE, Lee HS, Kim WH (2007). Prognostic significance of loss of c-fos protein in gastric carcinoma. Pathology oncology research.

[24] Zhou L, Zhang JS, Yu JC, Cui QC, Zhou WX, Kang WM, Ma ZQ (2010). Negative association of c-fos expression as a favorable prognostic indicator in gastric cancer. Archives of medical research.

[25] Li K, Cao RJ, Zhu XJ, Liu XY, Li LY, Cui SS (2015). Erythropoietin Attenuates the Apoptosis of Adult Neurons After Brachial Plexus Root Avulsion by Downregulating JNK Phosphorylation and c-Jun Expression and Inhibiting c-PARP Cleavage. Journal of molecular neuroscience.

[26] Haas S, Kaina B (1995). c-Fos is involved in the cellular defence against the genotoxic effect of UV radiation. Carcinogenesis.

[27] Pandey MK, Liu G, Cooper TK, Mulder KM (2012). Knockdown of c-Fos suppresses the growth of human colon carcinoma cells in athymic mice. International journal of cancer.

[28] Li S, Xu X, Xu X, Hu Z, Wu J, Zhu Y, Chen H, Mao Y, Lin Y, Luo J, Zheng X, Xie L (2013). MicroRNA-490-5p inhibits proliferation of bladder cancer by targeting c-Fos. Biochemical and biophysical research communications.

[29] Lan G, Yang L, Xie X, Peng L, Wang Y (2015). MicroRNA-490-5p is a novel tumor suppressor targeting c-FOS in human bladder cancer. Archives of medical science.

[30] Hartwell LH, Kastan MB (1994). Cell cycle control and cancer. Science (New York, NY).

[31] Smits VA, Medema RH (2001). Checking out the G(2)/M transition. Biochimica et biophysica acta.

[32] Kim H, Chen J (2008). New players in the BRCA1-mediated DNA damage responsive pathway. Molecules and cells.

[33] Seo HR, Lee DH, Lee HJ, Baek M, Bae S, Soh JW, Lee SJ, Kim J, Lee YS (2006). Cyclin G1 overcomes radiation-induced G2 arrest and increases cell death through transcriptional activation of cyclin B1. Cell Death Differ.

[34] Pruschy M, Shi YQ, Crompton NE, Steinbach J, Aguzzi A, Glanzmann C, Bodis S (1997). The proto-oncogene c-fos mediates apoptosis in murine T-lymphocytes induced by ionizing radiation and dexamethasone. Biochemical and biophysical research communications.

[35] Bai L, Mao R, Wang J, Ding L, Jiang S, Gao C, Kang H, Chen X, Sun X, Xu J (2015). ERK1/2 promoted proliferation and inhibited apoptosis of human cervical cancer cells and regulated the expression of c-Fos and c-Jun proteins. Medical oncology (Northwood, London, England).

[36] Shi R, Peng H, Yuan X, Zhang X, Zhang Y, Fan D, Liu X, Xiong D (2013). Down-regulation of c-fos by shRNA sensitizes adriamycin-resistant MCF-7/ADR cells to chemotherapeutic agents via P-glycoprotein inhibition and apoptosis augmentation. Journal of cellular biochemistry.

[37] Guo JC, Li J, Zhao YP, Zhou L, Cui QC, Zhou WX, Zhang TP, You L (2015). Expression of c-fos was associated with clinicopathologic characteristics and prognosis in pancreatic cancer. PloS one.

[38] Fan Q, He M, Deng X, Wu WK, Zhao L, Tang J, Wen G, Sun X, Liu Y (2013). Derepression of c-Fos caused by microRNA-139 down-regulation contributes to the metastasis of human hepatocellular carcinoma. Cell biochemistry and function.

[39] Liu G, Ding W, Liu X, Mulder KM (2006). c-Fos is required for TGFbeta1 production and the associated paracrine migratory effects of human colon carcinoma cells. Molecular carcinogenesis.

[40] Liu ZG, Liu L, Xu LH, Yi W, Tao YL, Tu ZW, Li MZ, Zeng MS, Xia YF (2012). Bmi-1 induces radioresistance in MCF-7 mammary carcinoma cells. Oncology reports.

[41] Gu X, Wang X, Xiao H, Ma G, Cui L, Li Y, Zhou H, Liang W, Zhao B, Li K (2015). Silencing of R-Spondin1 increases radiosensitivity of glioma cells. Oncotarget.

